# Cancer signs and risk factors awareness in Addis Ababa, Ethiopia: a population-based survey

**DOI:** 10.1186/s13027-022-00477-5

**Published:** 2023-01-04

**Authors:** Zinaye Tekeste, Nega Berhe, Mahlet Arage, Abraham Degarege, Yohannes Adama Melaku

**Affiliations:** 1 College of Medicine and Public Health, Flinders University, Bedford Park, SA Australia; 2 Aklilu Lemma Institute of Pathobiology, Addis Ababa University, P.O. Box 1176, Addis Ababa, Ethiopia; 3 Department of Epidemiology, College of Public Health, 984395 Nebraska Medical Center, University of Nebraska Medical Center, Omaha, NE USA; 4 Cancer Epidemiology Division, Cancer Council Victoria, Victoria, Australia

**Keywords:** Cancer awareness, Symptom awareness, Cancer, Risk factor awareness

## Abstract

**Background:**

There is a paucity of data on public awareness of cancer in Ethiopia. This study assessed cancer signs, symptoms, and risk factors awareness among individuals aged 18 and older in Addis Ababa, Ethiopia.

**Method:**

A population-based face-to-face interview was conducted applying a validated cancer awareness measure (CAM) tool. A total of 600 adults (315 males and 285 females) were recruited using a multistage sampling technique. One open-ended and ten closed-ended questions were used to assess awareness of cancer signs and symptoms. To assess awareness of cancer risk factors, one open-ended and twelve closed-ended questions were used. Logistic regression analysis was used to test the association between sociodemographic status and awareness of cancer signs, symptoms, and risk factors.

**Results:**

Based on the responses for the open-ended questions, unexplained bleeding (23.16%) and smoking (24.17%) were the most frequently recalled cancer sign and risk factor, respectively. Based on the responses for the closed questions, the majority of respondents identified tiredness all the time (80.7%) as a cancer symptom and alcohol use (82.5%) as a cancer risk factor.  The odds of cancer signs and symptoms awareness was higher in those with primary (AOR = 4.50, 95% CI, 1.72–11.79, *p* = 0.02), secondary (AOR = 4.62; 95% CI 1.86–11.43; *p* = 0.001), and tertiary (AOR = 7.51; 95% CI 3.04–18.56; *p* < 0.001) education than those who were illiterate. The odds of awareness about cancer signs and risk factors was 0.28 (95% CI 0.12–0.65; *p* = 0.003) and 0.22 (95% CI 0.83–0.58; *p* = 0.002) times lower, respectively, among individuals aged 60 and older than those aged 18 to 29.

**Conclusions:**

Young adults who attended formal education of primary or higher level may have a better cancer signs and symptoms awareness. Future education interventions to increase awareness of the society in Addis Ababa may target illiterate and the elderly.

## Background

Cancer is the leading cause of death worldwide [1]. In 2020, there were about 19.3 million new cancer cases and nearly 10.0 million cancer deaths worldwide [2]. With about 2.3 million new cases (11.7%), breast cancer is the most commonly diagnosed cancer in the world, followed by lung (11.4%), colorectal (10.0%), prostate (7.3%), and stomach (5.6%) cancers [2]. Cancer deaths and incidence are increasing globally, primarily due to aging, poor public awareness of cancer, population growth, and increased prevalence of cancer risk factors [2–4].

The burden of cancer has increased in Sub-Saharan Africa (SSA) [5]. In 2018, there were approximately 752,000 new cancer cases and 506,000 cancer deaths in SSA [6]. Breast cancer is the most common cancer in SSA, followed by cervical, prostate, colorectal, and liver cancers [2]. Cancer death and incidence are rapidly increasing in the region, and cancer deaths are expected to rise by 77% between 2010 and 2030 [7]. Similar to other SSA countries, cancer is becoming more common in Ethiopia [8]. The country's annual cancer incidence is estimated to be around 60,960 cases, with an annual mortality of over 44,000 [8, 9]. Breast cancer is the most common cancer in Ethiopia, accounting for 30.2% of all cases, followed by cervical (13.4%) and colorectal cancers (5.7%) [8, 9]. Despite Ethiopia's increasing cancer burden, cancer control has not been a national priority in recent decades [8, 10].

Moreover, Ethiopia, like many countries in the world, is experiencing rapid economic growth, urbanization, and increased globalization of unhealthy food and consumer goods markets [11, 12]. These changes increased the prevalence of modifiable cancer risk factors, such as alcohol and tobacco use in Ethiopia, expected to increase the burden of cancer in the country [11]. Some of the major risk factors for cancer are also prevalent in the country’s general population. For instance, Ethiopia is classified as a hepatitis endemic country by the World Health Organization, and over 10 million Ethiopians have been reported to live with hepatitis B virus [13–15]. Chronic hepatitis B and C viral infections are also reported to be responsible for over 60% of chronic liver diseases in the country [16].

Taking into account the possible effect of the aforementioned changes in the burden of cancer risk factors and other factors in increasing the burden of cancer in Ethiopia, the country developed a national cancer control plan in 2015, which was finalized in 2020, with the goal of reducing cancer incidence and mortality by 15% by 2020 [17]. Until 2020, the program implemented several strategies to achieve this goal. However, despite previous efforts, the country's cancer incidence and rate of late diagnosis are rising, while survival rates are declining [17]. Thus, improved cancer control efforts or plans are needed to promote early detection and improve outcomes.

Prevention and early detection are important components of cancer control, and improving cancer control efforts necessitate improvements in both [18, 19]. The general public's basic knowledge and awareness of cancer form the foundation for advancements in prevention and early detection [5]. Culture, religious beliefs, and socioeconomic status, however, have all been reported to influence public cancer awareness and knowledge [20–22]. Thus, determining the impact of these factors on public awareness of cancer contributes to the improvement of cancer control efforts.

Socioeconomic differences have been reported to influence the level of cancer signs, symptoms, and risk factors awareness in different countries [23]. People with a higher level of education in Saudi Arabia and Indonesia are more aware of cancer signs and symptoms than those who cannot read or write [24, 25]. In Denmark, economic status has been linked to differences in cancer risk factors awareness, with those with a high economic status having a higher level of awareness [26]. Women in the United Kingdom are more aware of cancer signs and symptoms than men [27]. A similar study conducted in the United Kingdom found age-related differences in cancer signs awareness [27].

In Ethiopia, little attention has been paid to examining the impact of culture, socioeconomic status, and religious beliefs on public awareness of cancer [28]. So far, few studies have been undertaken to examine public awareness of specific cancer types, such as cervical [29–31], breast [32–34], and colorectal cancers [35]. Moreover, more than 80% of cancer cases in Ethiopia are identified at an advanced stage, which is suggested to be due to a lack of public awareness of cancer signs and symptoms [28]. Thus, we assessed the socio-economic differences in awareness of cancer signs, symptoms, and risk factors among individuals aged 18 and older in Addis Ababa, Ethiopia’s capital city. To the best of our knowledge, this is the first study in Addis Ababa to use a validated measure to assess cancer awareness in a population-based sample.

## Methods

### Study setting

A cross-sectional survey was conducted in Addis Ababa, Ethiopia, in 2021. Addis Ababa has a population of 3,435,028 people (as of the 2017 population projection) and is divided into ten sub-cities [36]. Addis Ketema, Akaky Kaliti, Arada, Bole, Kolfe Keranio, Gulele, Lideta, Yeka, Kirkos, and Nifas Silk are its ten sub-cities. Residents of the sub-cities are ethnically and socioeconomically diverse [37, 38]. Adult literacy in Addis Ababa was about 80% for females and over 93% for males in 2007 [39].

### Sample size

The sample size was determined using Cochran's single proportion formula considering a 95% confidence interval, a 5% margin of error, and a proportion of 52.2% based on previous research [40, 41]. Due to multistage sampling, a design effect of 1.5 was assumed. With a 5% non-response rate, the total sample size was estimated to be 595. The study included 600 Addis Ababa residents aged 18 and older, and the response rate was 95.24%.

### Sampling technique

Multistage sampling was used in the survey. First, out of the ten sub-cites in Addis Ababa, four (Kolfe Keranio, Arada, Yeka, and Nifas Silk) were chosen by lottery. Then three Kebeles (the smallest administrative unit) were randomly chosen from each sub-city. The sample size was divided equally among the four Sub-cities because the number of households in the selected sub-cities was nearly the same [42]. Accordingly, 150 households were chosen from the three Kebele in each sub-city. To establish the direction and start of data collection, the spinning pen method was used in the center of each Keble. The gap between each selected household (sampling interval) was determined by dividing the total number of houses in each Kebele by the assigned sample size. The lottery approach was used to select one interviewee when there were two or more eligible members in a household. When an eligible participant was not available for an interview after several attempts, the home was permanently vacant, or the occupants decline to participate, the next closest house was visited. The household, Kebele, interviewee, and sub-cities were all manually chosen through lottery methods.

### Data collection

The Cancer Awareness Measure (CAM) questionnaire was utilized to assess awareness about cancer symptoms and risk factors. The CAM is a validated standard questionnaire developed by Cancer Research UK, University College London, King's College London, and Oxford University to measure awareness of cancer signs, symptoms, and risk factors [43, 44]. The questionnaire was translated into Amharic, the most widely spoken language in the study area. Forward and backward translation was done by independent individuals. Before the questionnaire was implemented, there was expert agreement on the Amharic and English versions. During the translation process, cultural appropriateness was maintained. The survey questionnaire translation team consists of experts familiar with the concepts the questionnaire intends to measure, language experts, and a naive translator who is unaware of the questionnaire's objective. Two experts also evaluated the questionnaire's clarity and relevance, and the questionnaire's content validity index was found to be 0.97. Experts also evaluated whether the questionnaire measures what it claims to measure; whether it is simple and easy to understand; whether any items are inappropriate, redundant, or missing; whether it is likely to address the research objective; whether it is relevant, flow, and arrangement; and whether it is wordy. A pilot test was also conducted with 50 people living in Kebeles (Kebele 01/18 in Lideta and 13/14 in Gulele Sub-cities) who were not part of the study sample and were not included in the actual survey. The pilot survey was conducted by the same survey team that conducted the final/actual survey. Any issues discovered with the questionnaire were addressed before conducting the actual survey.

A face-to-face survey was conducted to administer the CAM. Four interviewers, a data entry person, and a field coordinator were employed. The interviewers have at least two years of college education. A field coordinator with survey experience was hired to ensure that questionnaires were complete and legible and to resolve any surveyor performance issues that arose. One person was assigned to enter completed questionnaires into a survey database daily. Data collectors and supervisors were trained for two days on the data collection process, including data collection methods. Participation in the study was entirely voluntary, and participants gave their written consent to take part. By assuring respondents of confidentiality and anonymity, potential social desirability bias was reduced. Moreover, interviewer bias was reduced by training surveyors to appear neutral while collecting data.

There were one open-ended (recall) and ten closed-ended (recognize) questions about cancer signs and symptoms. The open-ended question was designed to see how many cancer signs and symptoms a respondent could recall without prompting. The close-ended questions were used to assess the awareness scale of signs and symptoms. The stem question for the closed-ended question was as follows: "The following may or may not be signs and symptoms for cancer. We are interested in your opinion." Following that is a list of ten cancer signs and symptoms: unexplained weight loss, unexplained lump/swelling, unexplained persistent pain, unexplained bleeding, persistent cough/hoarseness, difficulty in swallowing, sore that does not heal, coughing up blood, shortness of breath, and tiredness all the time. The response for each sign and symptom of cancer in the list were recorded as "Yes" (‘1’), "No" (0), and "I don't know". “I don’t know” responses were considered as ‘no’, and the total score was calculated by adding the response (1/0) for each of the ten signs and symptoms of cancer on the list. Hence, the total knowledge score for signs and symptoms of cancer ranged from 0 to 10.

Similarly, there were one open-ended and twelve closed-ended questions about cancer risk factors. The stem question for the closed-ended questions is as follows: "These are some of the things that can increase a person's chance of developing cancer. How much do you agree that each of these can increase a person's chance of developing cancer? "Following that is a list of twelve cancer risk factors: smoking, passive smoking, low intake of fruit/vegetables, alcohol consumption, overweight (BMI = 25.0–29.9), obese (BMI = 30.0 and above), sunburnt/exposure to the sun, older age, eating red or processed meat, stress, not doing enough exercise/physical activity, and family history of cancer/having a close relative with cancer. There were three response options: "Agree (‘1’)," "Disagree (‘0’)," and "Not sure (‘0’)." ‘‘Not sure’’ responses were considered as “disagree”, and the total score was calculated by adding the response (1/0) for each of the 12 cancer risk factors on the list [45]. The total knowledge score for the risk factors was obtained by summing the scores for the 12 risk factors of cancer in the list. To reduce bias, open-ended questions were asked before closed questions for both signs and risk factors.

### Data analysis

The data were cleaned and checked for outliers, errors, and omissions. The overall level of awareness about cancer signs and risk factors was classified as poor or good using the mean value as a cut-off point. Respondents with awareness scores higher than the mean, as previously reported, were considered to have good awareness [31]. The association between sociodemographic variables and the risk of cancer was determined using logistic regression models by estimating odds ratios (ORs) and 95% confidence intervals (CIs). Only complete samples were used for the logistic regression analysis. Moreover, monthly income was excluded from the logistic analysis because the majority of participants preferred not to disclose their income. Participants were divided into two groups based on their monthly income, using the Ethiopian average monthly income as a reference [46]. *p* values ≤ 0.05 were considered statistically significant. Statistical Package for the Social Sciences (SPSS) version 26.0 (SPSS Inc., Armonk, NY) was used to enter and analyze the data.

## Results

### General characteristics of the study participants

Table 1 shows the general characteristics of the study participants. The awareness of cancer signs and risk factors was assessed among 600 Addis Ababa residents aged 18 and older. The majority of the study participants (67%) were between the ages of 18 and 39. About 48% of participants were women, 48.66% were married, and 52.17% had completed post-secondary education. More than 30% of the participants had a monthly income of more than 5055 Ethiopian birr, and 20% were government employees.

**Table 1:** General characteristics of the study participants (N = 600)

Socio-demographic characteristics	Number	%
Age in years		
18–29	224	37.33
30–39	178	29.67
40–49	97	16.17
50–59	53	8.83
≥ 60	48	8.00
Gender		
Men	315	52.50
Women	285	47.50
Marital status		
Married	292	48.66
Single	247	41.17
Divorced	33	5.51
Prefer not to say	28	4.66
Education level		
Primary	61	10.17
Secondary	138	23.00
Diploma	105	17.50
Degree and above	208	34.67
Illiterate	42	7.00
Prefer not to say	46	7.66
Employment		
Governmental organization	120	20.00
Non-governmental organization/private	309	51.50
Retired	25	4.17
Unemployed	87	14.50
Prefer not to say	59	9.83
Monthly income		
> 5055 ETB	185	30.83
≤ 5055 ETB	147	24.50
Prefer not to say	268	44.67

### Awareness of signs and symptoms

Table 2 shows the awareness of cancer signs and symptoms measured by recognize (prompted) and recall (unprompted) questions. Without prompting, the most recalled sign was unexplained bleeding, which was recalled by 23.83% of the respondents. Unexplained weight loss was recalled by 23.16% of respondents. Approximately 23% and 13% of respondents recalled persistent cough and difficulty in swallowing, respectively. Only 3.5% and 0.83% of study participants recalled tiredness all the time and coughing up blood as cancer symptom and sign, respectively. For each cancer sign and symptom, awareness was lower when open questions were used than when closed questions were used.

**Table 2:** Percentage of participants who recalled and recognized cancer signs and symptoms (N = 600)

Signs and symptoms	Unprompted awareness	Prompted awareness
	Number (%) of respondents who recalled cancer signs and symptoms in response to open-ended questions	Number (%) of respondents who recognized cancer signs and symptoms in response to close-ended questions
Unexplained weight loss	139 (23.16)	414 (69)
Unexplained lump/swelling	7 (1.17)	460 (76.7)
Unexplained persistent pain	58 (9.67)	453 (75.5)
Unexplained bleeding	143 (23.83)	308 (51.3)
Persistent cough/hoarseness	138 (23.00)	209 (34.8)
Difficulty in swallowing	78 (13.00)	410 (68.3)
Sore that does not heal	4 (0.67)	426 (71)
Coughing up blood	5 (0.83)	248 (41.3)
Shortness of breath	35 (5.83)	465 (77.5)
Tiredness all the time	21 (3.5)	484 (80.7)

### Awareness of risk factor

Table 3 shows the study participants' awareness of cancer risk factors. Based on the responses for the open-ended questions, smoking was recalled by 24.17% of respondents as a cancer risk factor, followed by alcohol consumption (18%), old age (4.33%), and not doing enough exercise/physical activity (3.38%). Based on the responses for the closed questions, smoking was the most frequently recognized risk factor, with 86.33% of respondents recognizing it. 82.33% and 82.5% of respondents, respectively, recognied passive smoking and alcohol consumption as cancer risk factors. Only 34.5% of respondents identified being overweight as a risk factor for cancer.

**Table 3:** Percentage of participants who recalled and recognized cancer risk factors (N = 600)

Risk factors	Unprompted awareness	Prompted awareness
	Number (%) of respondents who recalled cancer risk factors in response to open-ended questions	Number (%) of respondents who recognized cancer risk factors in response to close-ended questions
Smoking	145 (24.17)	518 (86.33)
Exposure to another person's cigarette smoke (passive smoking)	2 (0.33)	494 (82.3)
Low intake of fruit/vegetables	0 (0.00)	360 (60)
Alcohol consumption	108 (18.00)	495 (82.50)
Overweight (BMI = 25.0–29.9)	17 (1.17)	207 (34.50)
Obese (BMI = 30.0 and above)	10 (1.67)	288 (48)
Sunburnt/exposure to the sun	10 (1.67)	310 (51.70)
Older age	26 (4.33)	365 (60.80)
Eating red or processed meat	3 (0.50)	429 (71.50)
Stress	17 (2.83)	290 (48.30)
Not doing enough exercise/physical activity	23 (3.83)	363 (60.50)
Family history/having a close relative with cancer	8 (1.33)	320 (53.30)

### Odds of cancer signs and risk factors awareness

Tables 4 and 5 show socioeconomic differences in the odds of cancer signs, symptoms, and risk factors awareness based on closed-question. When compared to illiterate respondents, those with primary (AOR = 4.50; 95% CI 1.72–11.79; *p* = 0.02), secondary (AOR = 4.62; 95% CI 1.87–11.42; *p* = 0.001), and tertiary (AOR = 7.51; 95% CI 3.04–18.56; *p* < 0.01) education were more likely to recognize cancer signs and symptoms. The odds of cancer signs and symptoms awareness were lower in those aged 60 and older (AOR = 0.28; 95% CI 0.12–0.68; *p* = 0.03) than in those aged 18 to 29. Those with primary education were 4.76 (95% CI 1.08, 21.03; *p* = 0.03) times more likely to be aware of cancer risk factors than those who were illiterate. Moreover, the odds of awareness about cancer risk factors was 0.22 (95% CI 0.83–0.58; p = 0.002) times lower among individuals aged 60 and older than those aged 18 to 29.

**Table 4:** Logistic regression results for factors associated with cancer signs and symptoms awareness in Addis Ababa, Ethiopia

Study variables	Level of awareness		COR [95% CI], *p* value	AOR [95% CI], *p* value
	Good n (%)	Poor n (%)		
Gender				
Male	209 (41.88)	108 (21.64)	I	I
Female	193 (38.68)	90 (18.04)	0.88, [0.61, 1.28], 0.51	1.20, [0.79, 1.81], 0.39
Marital status				
Single	165 (33.07)	83 (16.63)	I	I
Married	197 (39.48)	95 (19.04)	0.99, [0.68,1.46], 0.99	2.60,[0.91,7.39],0.07
Divorced	24 (4.81)	9 (1.80)	1.41, [0.57, 3.49], 0.45	0.86, [0.54, 1.37], 0.52
Education				
Illiterate	14 (2.81)	28 (5.61)	I	I
Primary	39 (7.82)	22 (4.41)	3.34, [1.43, 7.83], 0.005*	4.50, [1.72, 11.79],0.02*
Secondary	88 (17.64)	50 (10.02)	2.83, [1.33, 6.02], 0.007*	4.62, [1.86, 11.43], 0.001*
Tertiary	231 (46.29)	82 (16.43)	4.86, [2.39, 9.89], < 0.01*	7.51, [3.04, 18.56], < 0.01*
Employment				
Unemployed	57 (11.42)	30 (6.01)	I	I
Governmental organization	85 (17.03)	34 (6.81)	1.43, [0.77, 2.64], 0.24	0.46, [0.14, 1.49], 0.19
Non-governmental organization/private	206 (41.28)	102 (20.44)	0.97, [0.56, 1.62], 0.89	0.40, [0.14, 1.20], 0.10
Retired	16 (3.21)	9 (1.80)	0.85, [0.33, 2.18], 0.73	0.74, [0.24, 2.28], 0.59
Age in years				
18–29	148 (29.66)	77 (15.43)	I	I
30–39	129 (25.85)	48 (9.62)	1.49, [0.91, 2.43],0.11	0.51, [0.87, 2.48], 0.15
40–49	70 (14.03)	27 (5.41)	1.41, [0.78,2.54],0.26	0.21, [0.78,3.05], 0.21
50–59	37 (7.41)	16 (3.21)	1.27, [0.64,2.54],0.48	1.33, [0.62,2.88], 0.46
≥ 60	18 (3.6)	30 (6.01)	0.29, [0.15, 0.15], < 0.01*	0.28, [0.12, 0.65], 0.003*

OR, odds ratio; 95% CI, 95% confidence interval; AOR, adjusted odds ratio; COR, crude odds ratio

* Significant difference

**Table 5:** Logistic regression results for factors associated with risk factors awareness in Addis Ababa, Ethiopia

Study variables	Level of awareness		COR [95% CI], *p* value	AOR [95% CI], *p* value
	Good n (%)	Poor n (%)		
Gender				
Male	175 (35.07)	90 (18.04)	I	I
Female	161 (32.26)	73 (14.63)	0.75, [0.45, 1.25], 0.24	0.74, [0.37, 1.49], 0.41
Marital status				
Single	144 (28.86)	71 (14.23)	I	I
Married	172 (34.47)	85 (17.03)	0.66, [0.39,1.11], 0.11	1.31,[0.33,5.16], 0.70
Divorced	20 (4.01)	7 (1.40)	1.05, [0.29, 3.75], 0.94	1.16, [0.61, 2.21], 0.65
Education				
Illiterate	14 (2.81)	24 (4.81)	I	I
Primary	38 (7.62)	20 (4.01)	4.98, [1.22, 20.17], 0.02*	4.76, [1.08, 21.03], 0.03*
Secondary	76 (15.23)	46 (9.22)	1.04, [0.42, 2.53], 0.94	0.98, [0.34, 2.87],0.97
Higher Education	207 (41.48)	73 (14.63)	1.87, [0.79, 4.39], 0.15	1.53, [0.51, 4.58],0.45
Employment				
Unemployed	55 (11.02)	28 (5.61)	I	I
Governmental organization	84 (16.83)	30 (6.01)	1.58, [0.73, 3.40], 0.24	0.61, [0.12, 3.13],0.55
Non-governmental organization/private	182 (36.47)	96 (19.24)	1.56, [0.82, 2.97], 0.18	0.64, [0.14, 2.97],0.57
Retired	15 (3.01)	9 (1.80)	1.67, [0.44, 6.30], 0.44	0.56, [0.12, 2.62],0.46
Age in years				
18–29	99 (19.84)	34 (6.81)	I	I
30–39	55 (11.02)	20 (4.01)	0.71, [0.37, 1.35],0.29	0.74, [0.37, 1.49],0.41
40–49	35 (7.01)	14 (2.81)	0.72, [0.33,1.58],0.42	0.79, [0.33,1.91],0.61
50–59	16 (26.25)	28 (5.61)	6.00, [0.79, 45.66], 0.08	5.88, [0.73,46.67],0.09
≥ 60	131 (26.25)	67 (14.43)	0.20, [0.94, 0.42], < 0.01*	0.22, [0.83, 0.58],0.002*

OR, odds ratio; 95% CI, 95% confidence interval; AOR, adjusted odds ratio; COR, crude odds ratio

*Significant difference

## Discussion

In this study, we examined public awareness of cancer signs and risk factors among individuals aged 18 and older in Addis Ababa, Ethiopia. The study found that recall of cancer signs and risk factors using open questions was relatively low (< 30%) for all signs and risk factors. When closed questions were used, however, the recognition of cancer signs and risk factors was higher, with each sign and risk factor being identified by more than 30% of respondents. This was consistent with the findings of studies in Malesia [47] and the United Kingdom [27], in which recall of cancer signs and/or risk factors was lower with open-ended questions than with closed questions.

However, determining whether a score from an open-ended or closed-ended question better reflects cancer awareness is difficult. Since recall is limited by memory when open questions are asked, it understates awareness, and respondents may find it easy to guess when asked closed questions, resulting in an overestimation of awareness [27]. It has been suggested, however, that the accessibility of beliefs is important in predicting behavior, intentions, and attitudes, and that the most accessible beliefs are those that can be readily recalled or brought to mind: ‘people’s attitudes follow spontaneously and consistently from beliefs accessible in memory and then guide corresponding behavior’ [27]. When applied to our case, this concept suggests that recognizing signs and risk factors in response to closed-ended questions is less likely to result in help-seeking than recalling signs and risk factors in response to open-ended questions.

The demonstration that individuals aged 60 and older have low recognition of cancer signs and risk factors is in agreement with the report of Robb et al. [27] from the UK. In other studies, however, young age groups (15–34 years) were found to have lower cancer symptoms awareness [27, 48]. The Ethiopian government launched its first national cancer control plan in 2015, with one of its goals being to raise public awareness of cancer. The awareness program, however, gives little attention to the elderly [17]. This could explain why, in this study, older people were the least aware of cancer symptoms and risk factors, in contrast to previous studies.

Ethiopia's national cancer control plan also called for cancer awareness programs to be integrated into the daily routines of health education provided at all public healthcare facilities in the country [17]. Medication and transportation to the country's public healthcare facilities, on the other hand, are too expensive, which may have discouraged the elderly from seeking medical help or visiting the healthcare facilities and benefiting from the awareness program provided there [49–51]. This is primarily because the majority of Ethiopia's elderly do not have a steady source of income or adequate pensions [52, 53]. Moreover, poverty-related diseases such as HIV, tuberculosis, and malaria are on the rise in the country, leaving many children orphaned or abandoned [51, 54, 55]. Many elderly people are compelled to care for these children because they are almost always their grandchildren, exacerbating the older respondents' financial difficulties [51].

The Ethiopian government's free medical service system for the poor is also not functioning as intended for a variety of reasons [56, 57]. For instance, patients who visit public healthcare facilities; where free medical services for the poor are available, are frequently requested to purchase medications from private drugstores or be referred to private laboratories, which are more expensive than comparable public healthcare facilities [51]. This may also have discouraged old people in Ethiopia from seeking medical help or visiting public healthcare facilities and benefiting from cancer awareness education provided there.

In the present study, respondents with primary, secondary, and tertiary education were more aware of cancer signs and symptoms than those who were illiterate. This was consistence with the study in the UK [27] and Malesia [47], where a high level of education was associated with a higher cancer signs and syptoms awareness. Respondents with a high level of education in Denmark had also a higher level of cancer risk factors awareness [26]. In Ethiopia, particularly in Addis Ababa, government institutions and schools are adopting cancer awareness programs to teach students and staff how to lower their risk of developing cancer and recognize its early signs and symptoms [58, 59]. Moreover, educated people in Ethiopia use social media platforms more than illiterates [60], which provides them with more cancer information and raises their awareness of cancer symptoms and risk factors [61].

This study has strengths and limitations. It is the first study to assess cancer signs, symptoms, and risk factors awareness in Addis Ababa, Ethiopia, using a population-based sample. A second strength is that by ensuring participant confidentiality and anonymity, it reduced potential social desirability bias. It also reduced interviewer bias by training surveyors to appear neutral during the interview. The use of the CAM questionnaire, a validated measure of cancer awareness, is also the study's strength. One of the study's weaknesses is that the awareness scores for recognizing signs and risk factors in closed (recognize) questions may have been exaggerated because some participants may have guessed the correct answer. Moreover, awareness scores to open-ended (recall) questions may be underestimated because recall is limited by memory [27].

In conclusion, cancer signs awareness was lower in those aged 60 and older, as well as those who were illiterate. Individuals aged 60 and older also had a low awareness of cancer risk factors. Thus, the above-mentioned groups may benefit more from public health campaigns aimed at raising their awareness of cancer signs, symptoms and risk factors.

Although the Ethiopian government authorized the integration of cancer education into all public health facilities in the country [17], elderly people are less likely to benefit from such programs because financial constraints inhibited them from seeking medical help, visiting health institutions, and benefiting from cancer education provided in public health facilities [49–51].The government and other non-governmental organizations should collaborate to address the elderly's economic needs. This includes encouraging, and if necessary, engaging in and guiding income-generating activities for the elderly.

The finding that cancer signs and symptoms awareness is lower among illiterate people suggests that there is a need to raise awareness in this group. This includes focusing awareness campaigns on the illiterate and increasing the literacy level of city residents. Moreover, unlike educated people, illiterates in Ethiopia were found to use social media to a smaller extent [60]. However, using social media may help people become more aware of cancer signs and symptoms [61].  Thus, raising cancer awareness in illiterate people may be aided by using other methods of awareness creation, such as public cancer awareness creation via national television and radio, which are accessible to almost everyone.
