# Correction: Cancer signs and risk factors awareness in Addis Ababa, Ethiopia: a population-based survey

**DOI:** 10.1186/s13027-023-00538-3

**Published:** 2023-10-03

**Authors:** Zinaye Tekeste, Nega Berhe, Mahlet Arage, Abraham Degarege, Yohannes Adama Melaku

**Affiliations:** 1 College of Medicine and Public Health, Flinders University, Bedford Park, SA Australia; 2 Aklilu Lemma Institute of Pathobiology, Addis Ababa University, P.O.Box 1176, Addis Ababa, Ethiopia; 3 Department of Epidemiology, College of Public Health, 984395 Nebraska Medical Center, University of Nebraska Medical Center, Omaha, NE USA; 4 Cancer Epidemiology Division, Cancer Council Victoria, Victoria, Australia

**Correction to**: *Infectious Agents and Cancer* (2023) 18:1. 10.1186/s13027-022-00477-5

Following publication of the original article [1], the authors identified an error in the authorship: due to miscommunication, the last author (Amy Reynolds) did not have the opportunity to appropriately contribute to the manuscript, which violates the ICMJE criteria for authorship and her name therefore needed to be removed from the author group and the ‘Authors contributions’ section and her affiliation (original affiliation 5; Flinders Health and Medical Research Institute (Sleep Health)/Adelaide Institute for Sleep Health, College of Medicine and Public Health, Flinders University, Bedford Park, SA, Australia) needed to be removed from the list of affiliations.

In addition, the authors identified an error in the author name of the fifth author.

The incorrect author name is: Yohanns Melaku

The correct author name is: Yohannes Adama Melaku

The author group and the list of affiliations has been updated above and the original article [1] has been corrected.
